# Simultaneous Determination of Uric Acid and Caffeine by Flow Injection Using Multiple-Pulse Amperometry

**DOI:** 10.3390/bios13070690

**Published:** 2023-06-29

**Authors:** Ademar Wong, Anderson M. Santos, Maria H. A. Feitosa, Orlando Fatibello-Filho, Fernando C. Moraes, Maria D. P. T. Sotomayor

**Affiliations:** 1 Institute of Chemistry, São Paulo State University (UNESP), Araraquara 14801-970, SP, Brazil; 2 National Institute for Alternative Technologies of Detection, Toxicological Evaluation and Removal of Micropollutants and Radioactives (INCT-DATREM), Araraquara 14801-970, SP, Brazil; 3 Department of Chemistry, Federal University of São Carlos (UFSCar), São Carlos 13560-970, SP, Brazil

**Keywords:** uric acid, caffeine, FIA system, simultaneous determination, BDD, pulsed amperometry

## Abstract

The present study reports the development and application of a flow injection analysis (FIA) system for the simultaneous determination of uric acid (UA) and caffeine (CAF) using cathodically pretreated boron-doped diamond electrode (CPT-BDD) and multiple-pulse amperometry (MPA). The electrochemical profiles of UA and CAF were analyzed via cyclic voltammetry in the potential range of 0.20–1.7 V using 0.10 mol L^−1^ H_2_SO_4_ solution as supporting electrolyte. Under optimized conditions, two oxidation peaks at potentials of 0.80 V (UA) and 1.4 V (CAF) were observed; the application of these potentials using multiple-pulse amperometry yielded concentration linear ranges of 5.0 × 10^−8^–2.2 × 10^−5^ mol L^−1^ (UA) and 5.0 × 10^−8^–1.9 × 10^−5^ mol L^−1^ (CAF) and limits of detection of 1.1 × 10^−8^ and 1.3 × 10^−8^ mol L^−1^ for UA and CAF, respectively. The proposed method exhibited good repeatability and stability, and no interference was detected in the electrochemical signals of UA and CAF in the presence of glucose, NaCl, KH_2_PO_4_, CaCl_2_, urea, Pb, Ni, and Cd. The application of the FIA-MPA method for the analysis of environmental samples resulted in recovery rates ranging between 98 and 104%. The results obtained showed that the BDD sensor exhibited a good analytical performance when applied for CAF and UA determination, especially when compared to other sensors reported in the literature.

## 1. Introduction

Uric acid (UA) is the final decomposition product generated from the metabolism of purine (constituent of nucleic acids). It is present in the blood and can be detected in urine. The monitoring of UA in human serum is found to be essentially important, as the amount of UA present therein can be used as a relevant parameter for the diagnosis of many diseases including heart disease, Lesch–Nyhan syndrome, gout, diabetes, arthritis, rheumatic pain, and hyperuricemia [1]. The normal level of uric acid in urine is 2.0 mmol L^−1^ and may range between 0.12 and 0.45 mmol L^−1^ in the blood. To maintain a healthy level of uric acid, it is highly recommended that one avoids the excess consumption of purine-based foods (meat and seafood, sugar-sweetened beverages, and alcohol) [2,3].

Caffeine (CAF) is a xanthine alkaloid compound (1,3,7-trimethylxanthine), which is a naturally occurring substance found in the leaves, seeds, and fruits of various plant species; this compound is widely applied in pharmacological formulations, including analgesics and flu medication, and acts as stimulant in the central nervous system, where it increases alertness in addition to reducing sleepiness and improving short-term memory [4,5]. CAF can be found in chocolate, coffee, tea, cocoa, and in energy drinks. Excessive consumption of caffeine can provoke health disorders, such as irregular heartbeat, upset stomach, trembling hands, restlessness, diminished memory, asthma, and sleeping difficulty [6]. According to the FDA (Food Drug Administration), for health safety purposes, the amount of caffeine intake per day must not exceed 400 mg [7].

UA and CAF can be detected simultaneously in the human body, and the presence of these analytes above the recommended threshold or in excess can cause, as mentioned above, different health problems. These analytes can be found in biological matrices through food consumption and production in our body and in environmental matrices when disposed in the domestic sewage. Several studies reported in the literature have employed a wide range of methods for the determination of uric acid and caffeine; some of the methods applied have included the following: fluorescence [8], colorimetry [9], high-performance liquid chromatography (HPLC) [10], spectrophotometry [11,12], and electrochemical techniques [13,14]. In this context, Tasic et al. [15] published a review about recent advances in electrochemical sensors for caffeine determination. Likewise, Aafria et al. [16] and Lakshmi et al. [17] reported the use of electrochemical sensors and biosensors for the detection of uric acid in different media. Electrochemical techniques have the advantages of being simpler, cheaper, and more sensitive in addition to involving shorter analysis time compared to other traditional methods [18]. Thus, the application of an electrochemical technique in combination with flow injection analysis (FIA) provides us with relatively shorter time of analysis, greater sensitivity and selectivity, and operational simplicity in addition to low cost of analysis. The FIA technique has been applied for the determination of a wide range of compounds due to its high detection sensitivity, fast response, low reagent consumption, real-time monitoring capability, and operational simplicity. Compared to the voltammetric method, some of the main advantages of the amperometric method include real-time monitoring and the ability to analyze many samples at the same time. In addition, under the amperometric method, the contact time between the sample and the electrode surface is significantly reduced, and this helps minimize the adsorption effect [19,20].

The FIA system has been employed in combination with multiple pulse amperometry (FIA-MPA) for the simultaneous detection of analytes with different redox potentials. These analyses were primarily conducted using the activation potential, cleaning potential and redox potential of the analytes. Usually, under this type of analysis, boron-doped diamond (BDD) electrodes are often employed; these electrodes offer numerous analytical advantages, which include the following: better repeatability; ability to conduct analysis with negligible capacitive current; ease of operation/operational simplicity; low adsorption; and wide potential window [19,20]. In addition, BDD materials are found to possess sp^3^ hybridization, and this is one of the reasons for their high chemical and physical stability [20]. A number of studies published in the literature have reported the use of BDD electrodes for the determination of different analytes at micro and nanomolar levels [21,22].

In this work, we propose the use of a simple, highly sensitive and low-cost method for the simultaneous determination of uric acid (UA) and caffeine (CAF) in river water samples using a FIA-MPA system based on a cathodically pretreated BDD electrode. UA and CAF were chosen as matrices for the conduct of this study because these substances are commonly detected in the human body and are disposed of indiscriminately in the environment through domestic sewage.

## 2. Materials and Methods

### 2.1. Chemical 

Uric acid (UA) and caffeine (CAF) were purchased from Sigma Aldrich (Brazil). Potassium phosphate monobasic (KH_2_PO_4_), potassium phosphate dibasic (K_2_HPO_4_), sodium hydroxide (NaOH), and potassium chloride (KCl) were acquired from Synth (Brazil). UA and CAF solutions were prepared in basic and acid media, respectively, using ultrapure water. A 0.10 mol L^−1^ concentration sulfuric acid solution was prepared from stock solution, and a 0.10 mol L^−1^ phosphate buffer solution was prepared using a mixture of KH_2_PO_4_ and K_2_HPO_4_. Aqueous solutions were prepared using deionized water from the Milli Q system (resistivity > 18.2 MΩ cm).

### 2.2. Apparatus

The working electrode was a boron-doped diamond (BDD) film (8000 ppm) on a silicon wafer obtained from the Center Suisse de Electronique et de Microtechnique SA (CSEM), Neuchatêl, Switzerland. Voltammetric and amperometric experiments were performed using potentiostat/galvanostat (model PGSTAT-30, Autolab, Utrecht, The Netherlands) controlled by the GPES 4.9 software (Eco Chemie). 

HPLC-UV analysis was performed using Shimadzu^®^ Model 20A liquid chromatograph coupled with a UV/Vis detector and C18 column (250 × 4.6 mm, Shim–Pack CLC–ODS). The analysis was conducted under the following conditions: mobile phase: 40% methanol and 60% water (*v*/*v*); total running time for each analyte: 6 min; and detection wavelength: 275 nm [23,24].

### 2.3. Preparation of the BDD Electrode

Initially, the BDD electrode was subjected to pretreatment using a glass cell with three electrodes: Ag/AgCl/KCl (3.0 mol L^−1^) for the reference electrode, platinum wire auxiliary electrode, and BDD working electrode (with exposed area of 0.66 cm^2^), respectively. To render the electrode surface clean without impurities, the BDD electrode was washed with ultrapure water and ethanol mixture in an ultrasonic bath for 3 min. The anodic pretreatment (APT) and cathodic pretreatment (CPT) were performed using 0.50 mol L^−1^ H_2_SO_4_ solution and applied potentials of 0.04 A cm^−2^ and −0.04 A cm^−2^ consecutively for 30 s and 180 s, respectively. Subsequently, the BDD electrode was applied in the electrochemical cell for the amperometric and voltammetric analyses.

### 2.4. Preparation of Urine and River Water Samples

River water samples were collected from a river located in São Carlos, SP, Brazil (21°59′11.0′′ S 47°52′52.1′′ W) and stocked in amber flasks in a refrigerator at 0 °C. The samples were filtered, and 10 mL of the samples was enriched using two concentration levels of uric acid and caffeine. Synthetic urine samples were prepared using 0.040 mmol L^−1^ KCl, 0.040 mmol L^−1^ NH_4_Cl, 20 µmol L^−1^ CaCl_2_, 30 µmol L^−1^ KH_2_PO_4_, 30 µmol L^−1^ NaCl, and 0.040 mol L^−1^ urea in a 5.0 mL amber flask. To determine the concentration of these analytes, the samples prepared were injected into a FIA-MPA system using 0.10 mol L^−1^ sulfuric acid as electrolyte and the carrier solution. The results obtained from the application of the electrochemical and chromatographic methods were then compared. 

### 2.5. Analytical Procedure 

The BDD electrode was pre-treated anodically (APT–BDD) or cathodically (CPT–BDD) in 0.5 mol L^−1^ H_2_SO_4_ by applying 0.04 A cm^−2^ or −0.04 A cm^−2^ for 60 s or 180 s, respectively. The analytical response (electric current) of uric acid and caffeine was evaluated separately for the anodic and cathodic processes, as these analyzes were performed after optimizing the electrolyte solution, such as electrolyte and pH. On the other hand, the applied potentials for the oxidation of the analytes using the MPA were chosen through hydrodynamic voltammograms constructed from the threshold current data recorded at each applied potential pulse. Other variables involved in this method were systematically analyzed, such as flow rate, sample injection volume and pulsation time. In this sense, the calibration curves were obtained simultaneously from continuous injections of the support electrolyte containing the target analytes in the FIA-MPA system. The limit of detection (LOD) was obtained by processing experimental data of the signal-to-noise ratio in a 3-to-1 ratio. The accuracy of the analysis method proposed here was verified from several analysis repetitions (*n* = 15) for UA and CAF with two different concentrations. In addition, tests of the analytes in different matrices were performed to verify the efficiency of the detection method in real systems of environmental and biological complexity, such as river water and urine samples, in which such samples were enriched with several chemical compounds that are possible interferents in samples of this magnitude. Finally, river water and urine samples were added with known concentrations of UA and CAF and analyzed (*n* = 3) in terms of percentage recovery of the added concentration using the analytical curves constructed under optimized conditions.

## 3. Results and Discussion

### 3.1. Voltammetric Profile of UA and CAF

The analysis of the electrochemical profiles of UA and CAF was performed using an anodically pretreated BDD electrode (0.04 A cm^−2^ for 60 s) and a cathodically pretreated BDD electrode (−0.04 A cm^−2^ for 180 s); this was done in order to improve the performance of the BDD electrode in the redox reaction. The cathodic pretreatment (CPT) of the BDDE presents a predominantly hydrogen-terminated surface, while the anodic pretreatment (APT) of the BDDE presents a predominantly oxygen-terminated surface. Therefore, the detection can be strongly influenced by the pretreatment on the BDDE surface [25,26].

**Figure 1:**
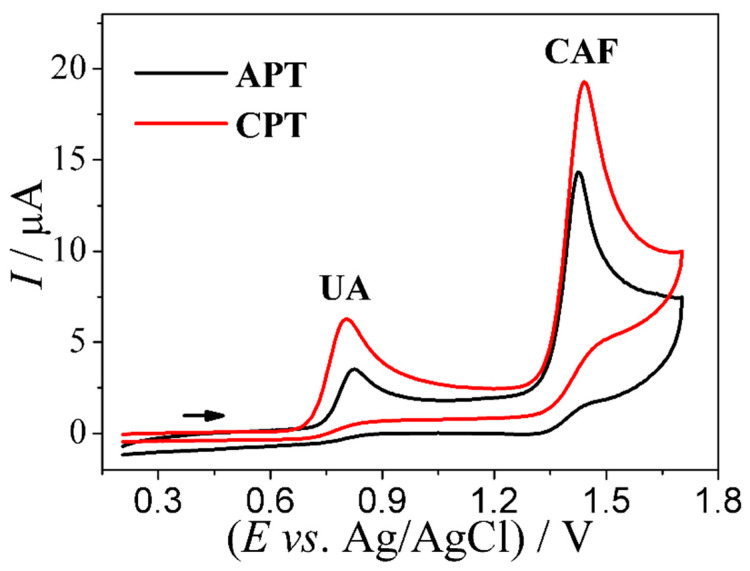
Using cyclic voltammetry for a comparative analysis of anodically (APT) and cathodically (CPT) pretreated BDD electrodes in a batch system based on the application of 5.0 × 10^−5^ mol L^−1^ UA and 5.0 × 10^−5^ mol L^−1^ CAF in 0.10 mol L^–1^ H_2_SO_4_ solution. Analysis conditions: v = 50 mV s^−1^, step potential = 4 mV, APT (0.04 A cm^−2^ for 60 s), and CPT (−0.04 A cm^−2^ for 180 s).

Figure 1 shows the cyclic voltammograms obtained from the application of 5.0 × 10^−5^ mol L^−1^ UA and 5.0 × 10^−5^ mol L^−1^ CAF solutions using the anodically (APT) and cathodically (CPT) pretreated BDD electrodes. Looking at Figure 1, one can clearly observe the low background current and oxidation peaks of the analytes with good peak separation at potentials of 0.80 V and 1.4 V for UA and CAF, respectively; apart from that, no reduction peaks are observed in the potential range of 0.20 V–1.7 V (v = 50 mV s^−1^ and step potential = 4 mV), which is typically characteristic of irreversible processes. The anodic peak current values obtained from the application of the APT-BDD and CPT-BDD electrodes for UA and CAF were 2.8 (UA) and 10.1 µA (CAF) for APT-BDD and 6.2 (UA) and 14.5 µA (CAF) for CPT-BDD. As can be seen, the CPT of the BDD electrode surface provided a significant increase in the magnitude of the oxidation peak current for both analytes, thus indicating that a predominantly hydrogen-terminated surface significantly improves the electrochemical activity of the BDD electrode for the processes of UA and CAF oxidation. In addition, the application of the BDD electrode modified with carbon materials (nanotubes and graphene) and metallic nanoparticles yielded no significant changes in the current signal. By virtue of that, the cathodically pretreated BDD electrode was selected and used for the conduct of further experiments.

### 3.2. Optimization of the FIA-MPA System

First, the cyclic voltammetry technique was used to evaluate the type of electrolyte and concentration that can be used for the conduct of electrochemical measurements. The electrolytes tested were as follows: phosphate buffer solution and sulfuric acid (H_2_SO_4_) solution. The results obtained from this analysis showed that the sulfuric acid solution exhibited better electrochemical response. Figures S1 and S2 in the Supplementary Material show the electrochemical responses of these electrolytes in the presence of UA and CAF and the concentration of the H_2_SO_4_ solution evaluated. Based on the results obtained from the analysis, the 0.10 mol L^−1^ H_2_SO_4_ solution was chosen for further experiments.

**Figure 2:**
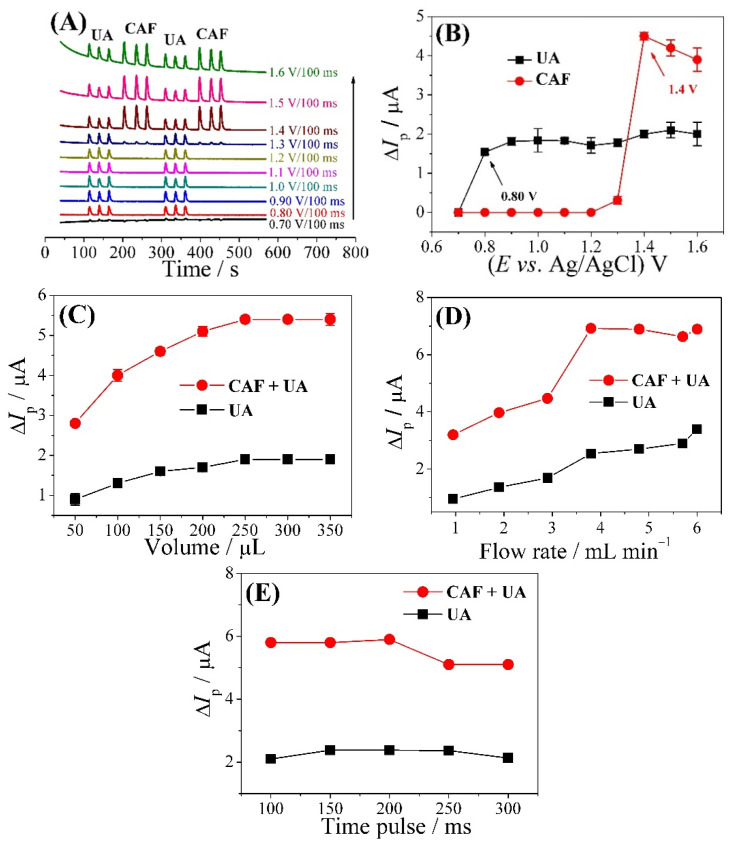
Optimization of the analytical parameters of the CPT-BDD electrode-based FIA-MPA system. (**A**) Amperograms obtained for injections UA and CAF solution and hydrodynamic voltammograms obtained by the values of peak current as a function of the (**B**) potential pulses applied, (**C**) injection sample volume: 50 to 350 µL, (**D**) flow rate: 0.95 to 6.0 mL min^−1^, and (**E**) pulse time: 100 to 300 ms. Analysis conditions: [UA] = 1.5 × 10^−5^ mol L^−1^; [CAF] = 1.5 × 10^−5^ mol L^−1^; electrolyte solution: 0.10 mol L^–1^ H_2_SO_4_.

The optimization of the FIA-MPA system was performed on the CPT-BDD electrode using 0.10 mol L^−1^ H_2_SO_4_ as the electrolyte solution. Figure 2A shows the amperograms based on the application of 10 sequential potential pulses (0.70 to 1.6 V), for which the best potential conditions were chosen from among the ten amperograms obtained. The magnitude of peak current for each potential pulse applied was measured and used to construct the hydrodynamic voltammogram for UA and CAF. As can be seen in the Figure 2B, UA starts to oxidize at low potentials (c.a. 0.70 V) and shows a current increase at 0.80 V. For CAF, the oxidation process starts c.a. 1.3 V and has a current increase of 1.4 V. Thus, potential pulses of 0.80 V and 1.4 V were selected, the first being for the oxidation of UA without interference from CAF and the second for when both compounds (UA and CAF) were oxidized and there is an oxidation stable for CAF.

After choosing the two best redox potentials, the injected sample volume (50 to 350 µL) (Figure 2C), flow rate (0.95 to 6.0 mL min^−1^) (Figure 2D), and pulse time (100 to 300 ms) (Figure 2E) were optimized in the FIA-MPA system experiments using a fixed concentration of UA and CAF of 1.5 × 10^−5^ mol L^−1^. The optimal values obtained were as follows: injection sample volume: 250 µL, flow rate: 3.8 mL min^−1^, and pulse time: 150 ms.

**Figure 3:**
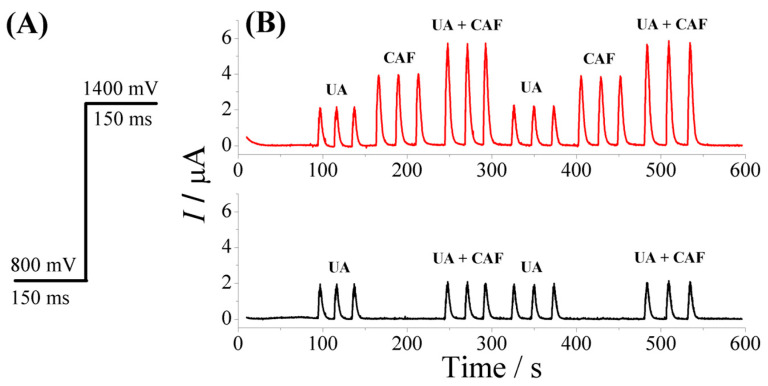
(**A**) MPA wave applied to the BDD working electrode as a function of time; (**B**) flow-injection pulse amperometric responses in triplicate for 1.5 × 10^−5^ mol L^−1^ UA and 1.5 × 10^−5^ mol L^−1^ UA and for UA + CAF at the same concentrations. Supporting electrolyte: 0.10 mol L^−1^ H_2_SO_4_; injection volume: 250 μL; and flow rate: 3.8 mL min^−1^.

Figure 3 shows the amperograms obtained with the application of two potential pulses (0.80 V (150 ms) and 1.4 V (150 ms)) for triplicate injections of 1.5 × 10^−5^ mol L^−1^ of UA, 1.5 × 10^−5^ mol L^−1^ of CAF, or a mixture of 1.5 × 10^−5^ mol L^−1^ of UA and CAF. The analysis of the amperograms at the potentials of 0.80 V and 1.4 V showed that only UA was oxidized at 0.80 V (in the presence or absence of CAF), while both analytes (UA and CAF) were oxidized at the potential of 1.4 V, that is, there was a sum of the magnitude of the signal of the oxidation process of UA and CAF. However, based on the results obtained, the UA oxidation current intensities do not equally apply the two potential pulses (0.80 V and 1.4 V), therefore, in view of the simultaneous determination of UA (0.80 V) and CAF (1.4 V), the use of a correction factor (CF) to obtain the exact value of the AU current at potential 1.4 V is necessary. The correction factor (CF) was calculated by dividing the magnitude of the current obtained at 1.4 V by the magnitude of the current obtained at 0.8 V (Equation (1)). Thus, the CF value (of 1.2 ± 0.1) obtained was useful for calculating the oxidation current of UA (1.4 V).

To obtain the actual concentration of CAF (1.4 V) in the simultaneous determination, we subtract the value of the oxidation current UA_(0.80 V)_ × CF from the total oxidation current at the potential of 1.4 V (UA + CAF), thus obtaining the value of the oxidation current only for CAF (Equation (2)). In this way, it is possible to quantify the two analytes simultaneously without interference.

(1) $$CF=\frac{I_{UA\;\left(1.4\;V\right)}}{I_{UA\;\left(0.80\;V\right)}}$$

(2) $$I_{CAF}=I_{total\;\left(1.4\;V\right)}-\left(CF\times I_{UA\;\left(0.80\;V\right)}\right)\;$$

### 3.3. FIA-MPA of Uric Acid and Caffeine

**Figure 4:**
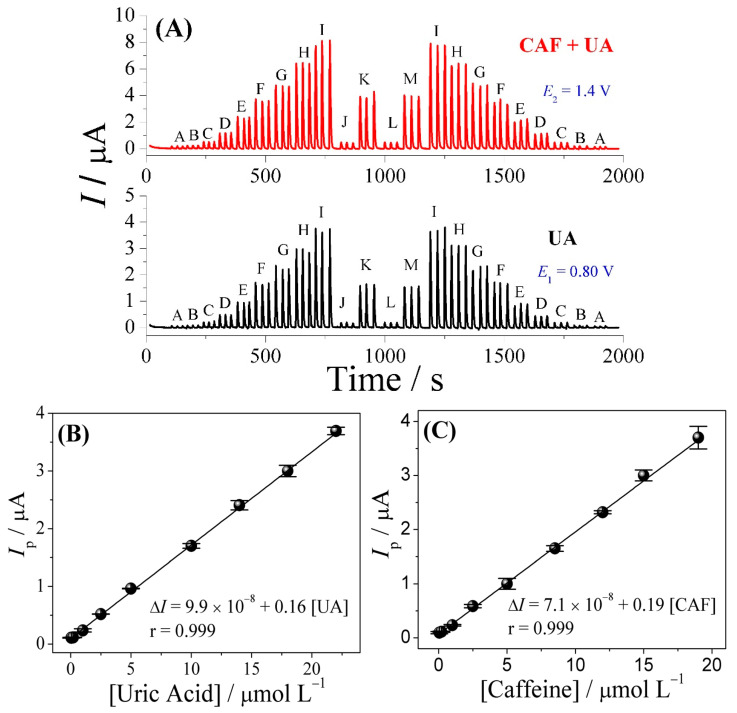
Fiagram obtained from the application of the CPT-BDD electrode, with the injection of UA at the following concentrations: (A–I) 5.0 × 10^−8^, 2.0 × 10^−7^, 1.0 × 10^−6^, 2.5 × 10^−6^, 5.0 × 10^−6^, 1.0 × 10^−5^, 1.4 × 10^−5^, 1.8 × 10^−5^, and 2.2 × 10^−5^ mol L^−1^, CAF at the following concentrations: (A–I) 5.0 × 10^−8^, 2.0 × 10^−7^, 1.0 × 10^−6^, 2.5 × 10^−6^, 5.0 × 10^−6^, 8.5 × 10^−6^, 1.2 × 10^−5^, 1.5 × 10^−5^ and 1.9 × 10^−5^ mol L^−1^, and (J–M) urine and river water samples fortified with different concentrations of UA and CAF (**A**). Analytical curve (*n =* 3) (inset) (**B**,**C**). Analysis conditions: supporting electrolyte/carrier solution: 0.10 mol L^−1^ H_2_SO_4_ solution; injection volume: 250 μL; and flow rate: 3.8 mL min^−1^.

Under optimized experimental conditions, the analytical curves for the simultaneous determination of UA and CAF were obtained and applied for the determination of UA and CAF in urine and river water samples. Figure 4A shows the amperograms obtained from the application of successive injections of different concentrations of the analytes in triplicate. The analytical curves were found to be linear in the concentration range of 5.0 × 10^−8^ to 2.2 × 10^−5^ mol L^−1^ for UA and 5.0 × 10^−8^ to 1.9 × 10^−5^ mol L^−1^ for CAF, with limits of detection (LOD) of 1.1 × 10^−8^ and 1.3 × 10^−8^ mol L^−1^, for UA and CAF, respectively (Figure 4B,C). The linear regression Equations (3) and (4) obtained for these analytes are given below:

UA curve: 9.9 × 10^−8^ + 0.16 ([UA] µmol L^−1^), r = 0.999 (3)

CAF curve: 7.1 × 10^−8^ + 0.19 ([CAF] µmol L^−1^), r = 0.999 (4)

In these analyses, UA and CAF standard solutions were injected in an increasing and decreasing concentration pattern, and the currents obtained at the same concentrations were found to be very similar; this outcome shows that the sensor did not exhibit any memory effects. 

A comparative analysis of the analytical performance of the electrochemical method proposed in this study with other methods reported in the literature showed that the proposed method exhibited better results in terms of linear concentration range and/or limit of detection (see Table 1) [27,28,29,30,31,32,33,34,35,36,37,38], especially when the comparison is centered on methods based on BDD electrodes [36,37,38]. Among the key advantages of the method proposed in this study include the following: (i) simpler electrochemical platform; (ii) better analytical performance with low linear concentration range; (iii) relatively lower limit of detection (at nanomolar level); and applicability for the analysis of different matrices.

The proposed method is also found to be of low cost and easy to operate in addition to exhibiting high baseline stability, good repeatability (see below), and the ability to perform 126 analytical determinations per hour.

**Figure 5:**
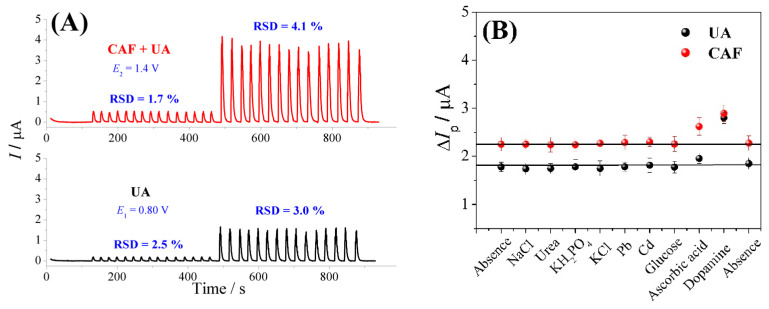
Analysis of repeatability of the CPT-BDD electrode-based FIA-MPA system using two concentration levels (1.0 × 10^−6^ and 1.0 × 10^−5^ mol L^−1^, respectively) of UA and CAF (**A**). Study of interference in the presence of different compounds (**B**). Analysis conditions: supporting electrolyte/carrier solution: 0.10 mol L^−1^ H_2_SO_4_ solution; injection volume: 250 μL; flow rate: 3.8 mL min^−1^; [UA] = 1.0 × 10^−5^; and CAF = 1.0 × 10^−5^ mol L^−1^.

The precision of the proposed FIA-MPA method was also evaluated through the repeatability technique using two different concentrations of UA and CAF (1.0 × 10^−6^ and 1.0 × 10^−5^ mol L^−1^) (*n =* 15), as shown in Figure 5A. The relative standard deviation (RSD) values obtained from this analysis ranged between 1.7 and 4.1%; this result showed that the proposed flow-injection method has good precision.

To further prove the suitability of the proposed method for the analysis of the analytes, electrochemical tests were performed in the presence of various interfering chemical compounds (Figure 5B) which are found in river water and urine samples (NaCl, urea, KH_2_PO_4_, KCl, Pb, Cd, glucose, ascorbic acid, and dopamine) in a concentration ratio of 1:1 (analyte:interfering molecule). The results obtained from these tests were found to be satisfactory; no significant effects were observed on the anodic peak current (*I*_pa_) intensities of the analytes after amperometric measurements with the exception of ascorbic acid and dopamine, which presented current values above the reference values for UA and CAF. The RSD values obtained for UA and CAF were less than 4.0%.

### 3.4. Application of the FIA-MPA Method in River Water and Urine Samples

The FIA-MPA method coupled with the CPT-BDD electrode was employed for the determination of UA and CAF in river water and urine samples, as shown in Table 2. The addition of UA and CAF in two concentration levels (8.0 × 10^−7^ and 8.0 × 10^−6^) resulted in recovery percentages ranging from 98 to 104%; clearly, this outcome points to low matrix effects. A comparative analysis of the results obtained from the application of the proposed flow-injection method and those obtained using the high-performance liquid chromatography (HPLC) reference method showed that the two methods exhibited very similar results; essentially, this shows that the proposed CPT-BDD electrode-based FIA-MPA method can be successfully applied for the simultaneous determination of UA and CAF considering that the relative error lies in the range of −2.5 to 1.2%.

The results obtained by the two methods also were statistically compared by applying the paired Student *t*-test (at a confidence level of 95%). Considering that the *t*_(experimental)_ values calculated for UA (0.33) and CAF (1.7) were smaller than the *t*_(critical)_ value (3.2), we can conclude that the results obtained using the two analytical methods did not present significant statistical difference at 95% confidence level. Hence, the here-proposed results demonstrated the precision of the FIA-MPA method and its effectiveness for the simultaneous determination of UA and CAF in river water and urine samples.

## 4. Conclusions

The results obtained from the cyclic voltammetry analysis conducted in the batch system showed that the cathodically pretreated BDD electrode exhibited a higher degree of efficiency compared to the anodically pretreated BDD electrode. A comparative analysis of the analytical performance of the proposed electrochemical method with other methods reported in the literature showed that the proposed method exhibited better results in terms of linear concentration range and/or limit of detection, especially when the comparison is centered on methods based on BDD electrodes. The CPT-BDD electrode-based FIA-MPA system proposed in this study exhibited good stability, satisfactory repeatability, and operational simplicity; apart from that, the technique was found to be extremely reliable and can be used for the conduct of numerous analyses per hour. The proposed method is also of low cost and has proven to be a highly efficient alternative method for the determination of UA and CAF with recovery rates close to 100% when applied in synthetic urine and river water samples. The findings of this study show that the proposed methodology is highly advantageous when compared to other analytical methods reported in the literature. 

## Figures and Tables

**Table 1:** Comparison of the analytical performance of electrochemical sensors for uric acid and caffeine determination.

Electrode	Analyte	Technique	Linear Range (µmol L^−1^)	LOD (µmol L^−1^)	Reference
n-HA/CPE ^a^	UA	DPV	0.068–50	0.05	[27]
Graphene-modified carbon fiber	UA	Amperometry	0.194–49.68	0.132	[28]
Poly(bromocresol purple)/GCE	UA	DPV	0.5–120	0.2	[29]
RGO−ZnO/GCE ^b^	UA	DPV	1.0–70.0	0.33	[30]
Poly(7A4HN2SA)/GCE ^c^	CAF	DPV	10–500	0.23	[31]
Nafion/GCE	CAF	DPV	0.99–10.6	0.79	[32]
ZnO/MWCNT/GCE ^d^	CAF	DPV	0.02–24.9	0.01	[33]
BiF-SPCE/CNFs ^e^	CAF	DPAdSV	0.20–1.0	0.05	[34]
FN/rGO-200/GCE ^f^	UA and CAF	DPV	4.0–21.5	1.3 and 1.6	[35]
BDD	UA and CAF	SWV	9.2–95.0 and 4.6–95.7	6.0 and 3.9	[36]
BDD	PCT and CAF	FIA-MPA	(0.050–1.3) × 10^3^ and (0.051–1.3) × 10^2^	0.66 and 0.87	[37]
BDD	CAF	BIA-MPA	10–1445	0.26	[38]
CPT-BDD	UA and CAF	FIA-MPA	0.050–22 and 0.050–19	0.011 and 0.013	This work

^a^ Nanoscale HA-modified GCE; ^b^ reduced graphene oxide (RGO)−ZnO nanocomposite-modified GCE; ^c^ 7-amino-4-hydroxynaphthalene-2-sulfonic acid on a glassy carbon electrode; ^d^ zinc oxide nanoparticles on multi-walled carbon nanotube-modified glassy carbon electrode; ^e^ bismuth film screen-printed carbon electrode nickel; ^f^ ferrite/reduced graphene oxide; DPV = differential pulse voltammetry; SWV = square wave voltammetry; BIA = batch-injection analysis; PCT = paracetamol.

**Table 2:** Determination of UA and CAF in urine and river water samples.

Samples	Analytes	Added/mol L^−1^	Comparative Method/mol L^−1^	Proposed Method/mol L^−1^	Recovery ** (Sensor, %)	Error *** %
			Found *	Found *		
River water	UA	8.0 × 10^−7^	(8.2 ± 0.1) × 10^−7^	(8.3 ± 0.1) × 10^−7^	104	+1.2
		8.0 × 10^−6^	(8.0 ± 0.1) × 10^−6^	(7.8 ± 0.1) × 10^−6^	98	−2.5
	CAF	8.0 × 10^−7^	(8.1 ± 0.1) × 10^−7^	(8.2 ± 0.1) × 10^−7^	102	+1.2
		8.0 × 10^−6^	(8.0 ± 0.2) × 10^−6^	(8.0 ± 0.1) × 10^−6^	100	0
Synthetic Urine	UA	8.0 × 10^−7^	(8.1 ± 0.1) × 10^−7^	(8.0 ± 0.1) × 10^−7^	100	−1.2
		8.0 × 10^−6^	(8.1 ± 0.2) × 10^−6^	(8.2 ± 0.1) × 10^−6^	102	+1.2
	CAF	8.0 × 10^−7^	(8.2 ± 0.2) × 10^−7^	(8.3 ± 0.4) × 10^−7^	104	+1.2
		8.0 × 10^−6^	(7.9 ± 0.2) × 10^−6^	(7.9 ± 0.3) × 10^−6^	99	0

* Average of 3 measured concentrations; ** recovery percentage = [Found/Added] × 100; *** Relative error = [(Proposed method−Comparative method)/(Comparative method)] × 100.

## Data Availability

Not applicable.

## Supplementary Materials

The following supporting information can be downloaded at: https://www.mdpi.com/article/10.3390/bios13070690/s1. Figure S1. Study of supporting electrolytes phosphate buffer and sulfuric acid at pH 4.5 using CTP-BDD electrode at v = 50 mV s^−1^. Figure S2. Effect of sulfuric acid concentrations (0.01; 0.05; 0.1 and 0.2 mol L^−1^) on the cyclic voltammograms using a CPT-BDD electrode and v = 50 mV s^−1^.
