# Supplementary material for: Simultaneous Determination of Uric Acid and Caffeine by Flow Injection Using Multiple-Pulse Amperometry
Source: Biosensors (Basel). 2023 Jun 29;13(7):690. doi: 10.3390/bios13070690 (PMC10377323; doi:10.3390/bios13070690)
Supplement: Supplementary file 1 [file biosensors-13-00690-s001.zip › biosensors-2463809-supplementary.pdf]

biosensors

SUPPLEMENTARY MATERIAL

Simultaneous Determination of Uric Acid and Caffeine by Flow Injection Using Multiple-Pulse Amperometry

Ademar Wong [1,2], Anderson M. Santos [3], Maria H. A. Feitosa [3], Orlando Fatibello-Filho [3], Fernando C. Moraes [3,*] and Maria D. P. T. Sotomayor [1,2]

1 Institute of Chemistry, São Paulo State University (UNESP), Araraquara 14801-970, SP, Brazil
2 National Institute for Alternative Technologies of Detection, Toxicological Evaluation and Removal of Micropollutants and Radioactives (INCT-DATREM), Araraquara 14801-970, SP, Brazil
3 Department of Chemistry, Federal University of São Carlos (UFSCar), São Carlos 13560-970, SP, Brazil
* Correspondence: fcmoraes@ufscar.br

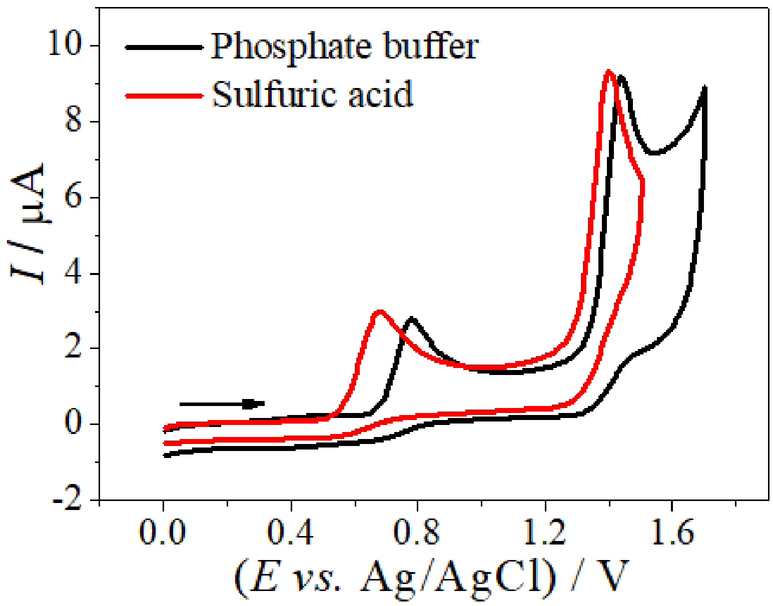

Figure S1. Study of supporting electrolytes phosphate buffer and sulfuric acid at pH 4.5 using CTP-BDD electrode at υ = 50 mV s^{-1}.

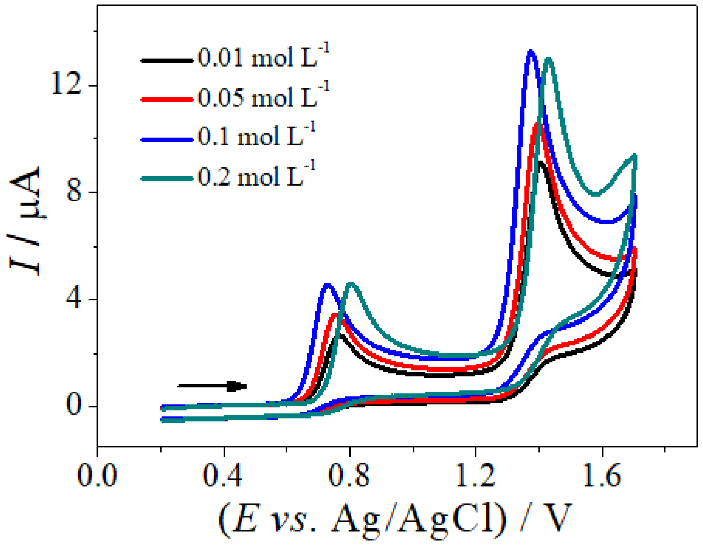

Figure S2. Effect of sulfuric acid concentrations (0.01; 0.05; 0.1 and 0.2 mol L^{-1}) on the cyclic voltammograms using a CPT-BDD electrode and υ = 50 mV s^{-1}.
